# MiR5651, miR170-3p, and miR171a-3p Regulate Cadmium Tolerance by Targeting *MSH2* in *Arabidopsis thaliana*

**DOI:** 10.3390/plants14132028

**Published:** 2025-07-02

**Authors:** Xianpeng Wang, Hetong Wang, Xiuru Sun, Zihan Tang, Zhouli Liu, Richard A. Ludlow, Min Zhang, Qijiang Cao, Wan Liu, Qiang Zhao

**Affiliations:** 1 Liaoning Key Laboratory of Urban Integrated Pest Management and Ecological Security, College of Life Science and Bioengineering, Shenyang University, Shenyang 110044, China; wxp19980807@163.com (X.W.); tangzhget@163.com (Z.T.); zlliu@syu.edu.cn (Z.L.); himoli12340@163.com (M.Z.); caojiang2010@126.com (Q.C.); 2 Key Laboratory of Ministry of Agriculture and Rural Affairs of Soybean Mechanized Production, College of Agriculture, Heilongjiang Bayi Agricultural University, Daqing 163000, China; sunxiuru2023@163.com; 3 Northeast Geological S&T Innovation Center, China Geological Survey, Shenyang 110034, China; 4 School of Biosciences, Cardiff University, Sir Martin Evans Building, Museum Avenue, Cardiff CF10 3AX, UK; ludlowra@cardiff.ac.uk; 5 College of Agriculture and Biological Sciences, Dehong Normal University, Mangshi 678400, China; liuwan63@163.com

**Keywords:** Cd stress, cell cycle, DNA damage response, DNA mismatch repair

## Abstract

The DNA mismatch repair (MMR) system plays a crucial role in repairing DNA damage and regulating cell cycle arrest induced by cadmium (Cd) stress. To elucidate the mechanism by which miRNAs target *AtMSH2* in regulating *Arabidopsis*’ response to Cd stress, the wild-type *Arabidopsis*, *Atmsh2* mutant, and three miRNA-overexpressing transgenic lines were grown hydroponically in half-strength MS solution containing cadmium (Cd) at concentrations of 0, 0.5, 1, 2, and 3 mg/L for 5 days. miRNA-seq analysis, bioinformatics prediction, dual-luciferase reporter assays, and qRT-PCR results demonstrated that miR5651, miR170-3p, and miR171a-3p specifically targeted *AtMSH2* and their expression levels showed a significant negative correlation. Compared to wild-type (WT) *Arabidopsis*, Cd stress tolerance was significantly enhanced in miRNA-overexpressing transgenic lines. Moreover, exogenous application of these three miRNAs in half-strength MS liquid medium also markedly improved Cd stress tolerance in wild-type *Arabidopsis*. Furthermore, the expression of these three miRNAs expression was further upregulated by Cd stress in a dose-dependent manner. Additionally, DNA damage response in miRNA-overexpressing transgenic lines was promoted based on the expression of DNA repair, DNA damage signaling, and cell cycle genes, which differed from both wild-type and *Atmsh2* plants. Taken together, miR5651, miR170-3p, and miR171a-3p participated in Cd stress response and improved plant Cd tolerance by mediating the expression of *AtMSH2*. Our study provides novel insights into the epigenetic mechanisms of Cd tolerance in plants, which sheds light on breeding for stress resilience in phytoremediation.

## 1. Introduction

Cadmium (Cd) pollution is a severe and urgent global environmental issue. Due to its wide range of sources and strong bioaccumulation characteristics, Cd has emerged as one of the most critical heavy metal pollutants in agricultural soil, which is ranked as the 4th most hazardous inorganic substance by the Agency for Toxic Substances and Disease Registry [1,2,3]. Among the limited number of effective remediation techniques for Cd-contaminated soils, phytoremediation—particularly based on phytoextraction using hyperaccumulators—has been extensively validated and widely recognized as an eco-friendly and sustainable approach [4,5,6]. Nonetheless, Cd is one of the most toxic heavy metals faced by plants. Although it is a non-essential element for plant growth and development, its high mobility in soils allows it to be easily absorbed by plant roots through cation and sulfate transporters and subsequently bioaccumulated in the human food chain, leading to severe health risks such as kidney dysfunction, osteoporosis, and cancers [7,8,9,10,11,12,13]. Exposure to Cd leads to multiple forms of DNA damage including base substitutions, base–base mismatches, insertion/deletion loops, DNA adducts, DNA breaks, DNA methylation, and DNA-strand cross-links [14,15]. In order to maintain genome integrity and prevent DNA damage transmission to daughter cells, the DNA mismatch repair (MMR) system is crucial for base–base mismatches, insertion/deletion loops, interstrand cross-links (ICLs), and double-strand breaks (DSBs), which triggers DNA damage response (DDR) to mediate cell cycle by activating ATR/ATM [16,17,18,19,20]. Thus, the efficacy of the DNA MMR system is considered as a proxy of Cd stress response, through regulating Cd-induced DNA damage sensitivity [21]. Moreover, *MSH2*, a key component of the MMR system, forms heterodimeric MutS complexes with MSH6, MSH3, and MSH7 in plants, which are responsible for the recognition of base–base mismatches, insertion/deletion loops, interstrand cross-links (ICLs), single-strand breaks (SSBs), and double-strand breaks (DSBs) with the replication protein A (RPA) complex and the MRE11-RAD50-NBS1 (MRN) complex [16,22,23].

MicroRNAs (miRNAs), 19–22 nt non-coding RNAs discovered in *Caenorhabditis elegans* and ubiquitously present in plants and animals, are processed from stem–loop regions of longer RNA transcripts to regulate gene expression at the post-transcriptional level by binding to specific sequences on target mRNAs, leading to mRNA cleavage or translational repression [24]. This mechanism enables miRNAs to critically modulate cellular processes such as differentiation, proliferation, and apoptosis [25,26]. Due to their indispensable regulatory role in living organisms, miRNAs have been considered as a molecular biomarker for various diseases and stress responses in plants [27,28,29,30,31,32]. In plants, upregulated or downregulated miRNAs induced by stress exert their critical physiological regulatory functions by either downregulating negative regulator target genes or upregulating positive regulator target genes during the stress response, thereby mitigating the toxic effects [33,34,35,36]. Due to recent progress in plant molecular biology, miRNAs have emerged as promising crop improvement players, given that miRNAs involve in plant vegetative growth, flowering, senescence, and fruit/grain setting [37,38,39]. Moreover, miRNAs have the potential as strategic tools for breeding stress-resilient crops. For example, miR393 and miR156 can improve salt and drought stress tolerance by targeting *TIR1* and *SPL* genes, respectively, while miR166 and miR395 are capable of controlling plant Cd accumulation by targeting ABC transporters and sulfate transporters to mediate Cd transmembrane transport [40,41,42]. With regard to the epigenetic regulation of the DNA MMR system, miR-21, miR-137, and miR-155 have been found in human colorectal cancer targeting 3′ UTR regions of *MSH2* or *MSH6* mRNAs, thereby suppressing MMR function [43,44,45]. Although miRNAs mediating DNA MMR function have been reported, only limited information is available for miRNAs targeting MMR genes, especially in plants.

In this study, we employed bioinformatics prediction to preliminarily screen candidate miRNAs targeting the *AtMSH2* gene. Subsequently, a tobacco (*Nicotiana benthamiana* L.) dual-luciferase reporter system was constructed to validate the targeting interaction between *MSH2* and candidate miRNAs in vitro. To further confirm this regulatory relationship in vivo, transgenic *Arabidopsis* lines overexpressing the candidate miRNAs were generated. Finally, under Cd stress conditions, we analyzed the expression dynamics of the relevant miRNAs in wild-type (WT) *Arabidopsis* and miRNA-overexpressing lines, thereby elucidating miRNAs targeting *AtMSH2* mediate Cd stress responses.

## 2. Materials and Methods

### 2.1. Materials, Growth, and Treatment Conditions

*Arabidopsis thaliana* (Columbia ecotype) and *Atmsh2* T-DNA insertion mutant lines (SALK_002708, the background of the lines is from Col-0), and tobacco seeds were provided by the Soybean High Yield Cultivation Technology Innovation Team at the College of Agronomy, Heilongjiang Bayi Agricultural University, Daqing, China.

About 500 *Arabidopsis* seeds were placed in a 2 mL centrifuge tube and surface-sterilized using the 1 mL of hypochlorite (10% *v*/*v*) followed by 1 mL of ethanol (75% *v*/*v*) for 3 min, then washed with sterile distilled water 5 times. The seeds were immersed in 1 mL sterile water and vernalized at 4 °C for 24 h. The seeds were then sown in a culture bottle containing 150 mL of sterilized half-strength MS medium [46] (Basal Salt Mixture, Caisson, Colorado Springs, CO, USA) with 1.5% (*w*/*v*) sucrose (pH 5.8). After years of extensive toxicological screening, our research group determined that the Cd stress concentration range was 0–3 mg/L [18,21]. This range encompasses low-to-high stress levels suitable for investigating miRNA-mediated cadmium responses. Concentrations exceeding 3 mg/L caused elevated *Arabidopsis* mortality, compromising consistent sampling; whereas concentrations below 0.5 mg/L were insufficient to induce the stress required for toxicity/tolerance studies. For the Cd treatment, 0 (the control), 0.5, 1, 2, and 3 mg/L Cd^2+^ in the form of CdCl_2_·2H_2_O (analytical grade with purity 99.5%, China) were added into the half-strength MS medium [46] solution. *Arabidopsis* seeds were placed in a climate chamber at 21 ± 1 °C under 12 h light/12 h dark for 5 days.

For the exogenous miRNA treatments, 50 seeds of *Arabidopsis* were sown in six-well plates containing 2 mL of liquid MS medium with 0, 1, or 3 mg/L Cd treatment and 500 ng synthetic ds-miRNAs. The six-well plates were placed on a continuous shaker at 21 ± 1 °C and 12 h light/12 h dark photoperiod for 5 days. All treatments and analyses were repeated in three independent replicates.

### 2.2. Bioinformatics Prediction of miRNAs Targeting the AtMSH2

The CDs sequence information of the *AtMSH2* gene was obtained from the NCBI database. The candidate miRNAs targeting the *AtMSH2* were selected using psRNATarget (https://www.zhaolab.org/psRNATarget/, accessed on 7 August 2017) with the parameters set as Expectation (E) < 2. The *Arabidopsis* miRNA sequence information was retrieved from the miRBase database (http://www.mirbase.org/, accessed on 7 August 2017).

### 2.3. Plant Expression Vectors Construction and Dual-Luciferase Analysis

Plant expression vectors containing the precursor sequences of candidate miRNAs (miR5651, miR170-3p, and miR171a-3p) were, respectively, constructed using the pGreen_GUS_competitor plasmid (Addgene ID 55208) following the method described by Liu et al. [47] and designated OE-miR5651, OE-miR170-3p, and OE-miR171a-3p. The plant expression vectors of 3′UTR-*AtMSH2* (containing the *AtMSH2* fragment targeting the candidate miRNAs), 3′UTR-P (the positive control, completely complementary to the candidate miRNA target sequences), and 3′UTR-N (the negative control, completely non-complementary to the candidate miRNA target sequences) were constructed using the pGreen_3′UTR_sensor (Addgene ID 55206) plasmid, respectively. The primer information is shown in Table S1.

Following the method described by Liu et al. [47], *Agrobacterium tumefaciens* containing 3′UTR_sensor (3′UTR-*AtMSH2*, 3′UTR-P, and 3′UTR-N) was simultaneously injected into tobacco plant leaves (with a leaf age of 6 weeks), respectively, with the *A. tumefaciens* containing GUS_sensor (OE-miR5651, OE-miR170-3p, and OE-miR171a-3p). The tobacco plants were cultivated in a climate chamber at 27 ± 1 °C and a 16 h light/8 h dark photoperiod. After 3 days of growth, the tobacco leaves injected with *A. tumefaciens* were harvested. The fluorescence intensities of firefly (*Lampyridae*) luciferase and *Renilla reniformis* luciferase in tobacco leaves were detected using the Dual-Luciferase Assay Kit reagents (Promega Corporation, Madison, WI, USA. catalog# E4550) on a full-wavelength multifunctional microplate reader (Thermo Fisher Scientific Inc, Waltham, MA, USA). The dual-luciferase analysis had three biological replicates. Each biological replicate had three technical replicates.

### 2.4. miRNA Overexpression Transgenic Arabidopsis Plants Construction

miRNA overexpression transgenic *Arabidopsis* plants (OE-miR5651, OE-miR170-3p, and OE-miR171a-3p) were transformed using the inflorescence infection method following the method of Cheng et al. [48]. The background of the transgenic *Arabidopsis* plants is Col-0. *A. tumefaciens* containing the GUS_sensor (OE-miR5651, OE-miR170-3p, and OE-miR171a-3p) was used. The transgenic *Arabidopsis* plants were screened with 20 mg/L glufosinate-ammonium. DNA was extracted from the plants for PCR detection of the *Bar* gene. Homozygous transgenic *Arabidopsis* plants were cultivated to the T3 generation for subsequent experiments.

### 2.5. RNA Extraction, First-Strand cDNA Synthesis, and qRT-PCR Analysis

The total RNA was extracted from 0.1 g fresh samples preserved at −80 °C using TransZol Plant (TRANS, Beijing, China) following the manufacturer’s instructions. The RNA concentration was detected using the ultramicrospectrophotometer NanoDrop 2000C (Thermo Fisher Scientific, Waltham, MA, USA). The first strand of cDNA was synthesized from 1 μg total RNA using the HiScript^®^ III RT SuperMix for qPCR (Vazyme Biotech, Nanjing, China) following the manufacturer’s instructions. The qRT-PCR analysis was performed using the ChamQ Universal SYBR qPCR Master Mix (Vazyme Biotech, Nanjing, China) on the ABI Step One™ + real-time PCR system (ABI, Waltham, MA, USA). The *UBQ 10* gene was used as the reference gene for signal normalization. The primers used for qRT-PCR are listed in Table S1. Relative gene expression levels between different treatments were calculated using the calculation method 2^−∆∆CT^ [49]. The qRT-PCR experiments had three biological replicates. Each biological replicate had three technical replicates.

### 2.6. miRNAs Expression Analysis

The miRNAs were extracted from 0.1 g fresh samples preserved at −80 °C using the TaKaRa MiniBEST Plant RNA Extraction Kit (Takara Bio Inc, Kusatsu-shi, Japan). The miRNA first strand was synthesized using the miRNA 1st Strand cDNA Synthesis Kit (by stem–loop) (Vazyme Biotech, Nanjing, China). The miRNA expression level was detected using the miRNA Unimodal SYBR qPCR Master Mix fluorescence quantitative detection kit (Vazyme Biotech, Nanjing, China) on the ABI Step One™ + real-time PCR system (ABI, Waltham, MA, USA). The *U6* gene was used as the reference gene for signal normalization. The primers used for miRNA expression analysis were listed in Table S1. Relative expression levels of miRNAs between different treatments were calculated using the calculation method 2^−∆∆CT^ [49]. The qRT-PCR experiments had three biological replicates. Each biological replicate had three technical replicates.

### 2.7. Statistical Analysis

All the experimental data were analyzed using SPSS (version 29.0) and reported as the mean ± SD (standard deviation) values. Different letters indicate statistically significant differences between treatments at *p* < 0.05 by one-way ANOVA with Tukey’s test. The figures were produced using GraphPad Prism10.

## 3. Results

### 3.1. Bioinformatics Prediction Results of miRNAs Targeting the AtMSH2

To obtain *AtMSH2* targeting miRNAs, the CDs sequence of the *AtMSH2* gene was searched using the NCBI database and analyzed using the psRNA Target website. Expectation (E) < 2 was set to achieve a higher credibility. cDNA library selection: “*Arabidopsis thalian*, transcript, removed miRNA gene, TAIR, version 10” “released on 2010_12_14” miRNAs targeting the *AtMSH2* gene were screened, and the matching results with the *AtMSH2* gene are shown in Table 1. Three candidate miRNAs were obtained through bioinformatics analysis techniques, namely miR5651, miR170-3p, and miR171a-3p.

### 3.2. Determination of the Targeting Relationship Between miRNAs and AtMSH2

**Figure 1:**
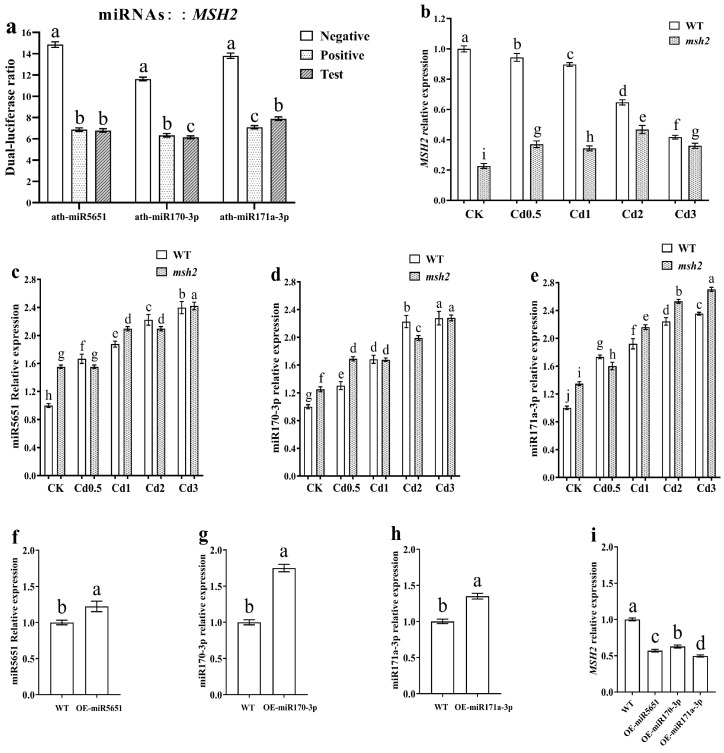
miR5651, miR170-3p, and miR171a-3p target and negatively regulate *AtMSH2*. The relative dual-luciferase activity in tobacco leaves (**a**): the three experimental groups shown from left to right correspond to miR5651, miR170-3p, and miR171a-3p—each co-infiltrated with their respective 3′ UTR expression vectors. The 3′ UTR vectors comprise negative control, positive control, and test constructs. “Positive Control” contains oligonucleotide DNA perfectly matching miRNAs, while “Negative Control” contains oligonucleotide DNA completely mismatched to miRNAs. The effect of Cd stress on the relative expression levels of *AtMSH2* (**b**), miR5651 (**c**), miR170-3p (**d**), and miR171a-3p (**e**) in the WT and *Atmsh2*. The relative expression levels of miR5651 (**f**), miR170-3p (**g**), miR171a-3p (**h**), and *AtMSH2* (**i**) in the miRNA overexpression transgenic *Arabidopsis* plants under control conditions, respectively. The gene expression level in the control (WT-CK) were set to 1 as the normalization for qRT-PCR analysis using the calculation method 2^−ΔΔCt^. Data are shown as mean ± SD of three independent experiments, and each biological replicates had three technical assays. Different letters indicate statistically significant differences between treatments at *p* < 0.05 by one-way ANOVA with Tukey’s test.

Dual-luciferin analysis was used to verify the targeting relationship between miR5651, miR170-3p, miR171a-3p, and *AtMSH2*. Compared with the negative control group, the relative luciferase activity in the miR5651, miR170-3p, and miR171a-3p test groups was significantly decreased, and showed no significant difference with the positive control group (Figure 1a). The qRT-PCR results demonstrated that Cd stress significantly downregulated the expression level of the *AtMSH2* gene in WT, while upregulated the expression levels of miR5651, miR170-3p, and miR171a-3p when compared with the control (Figure 1b–e). Furthermore, these effects exhibited a dose-dependent relationship with the Cd concentration. In *Atmsh2* mutants, Cd stress dose-dependently upregulated the expression of *AtMSH2*, miR5651, miR170-3p, and miR171a-3p.

In the miR5651, miR170-3p, and miR171a-3p overexpression transgenic *Arabidopsis* plants, the expression levels of miR5651, miR170-3p, and miR171a-3p were significantly upregulated, respectively. At the same time, the expression levels of *AtMSH2* in those miRNA-overexpressing transgenic plants were significantly decreased (Figure 1f–i). Taken together, miR5651, miR170-3p, and miR171a-3p can target and negatively regulate the expression of *AtMSH2*.

### 3.3. The Effect of miRNAs Targeting and Regulating AtMSH2 on Plant Growth Under Cd Stress

**Figure 2:**
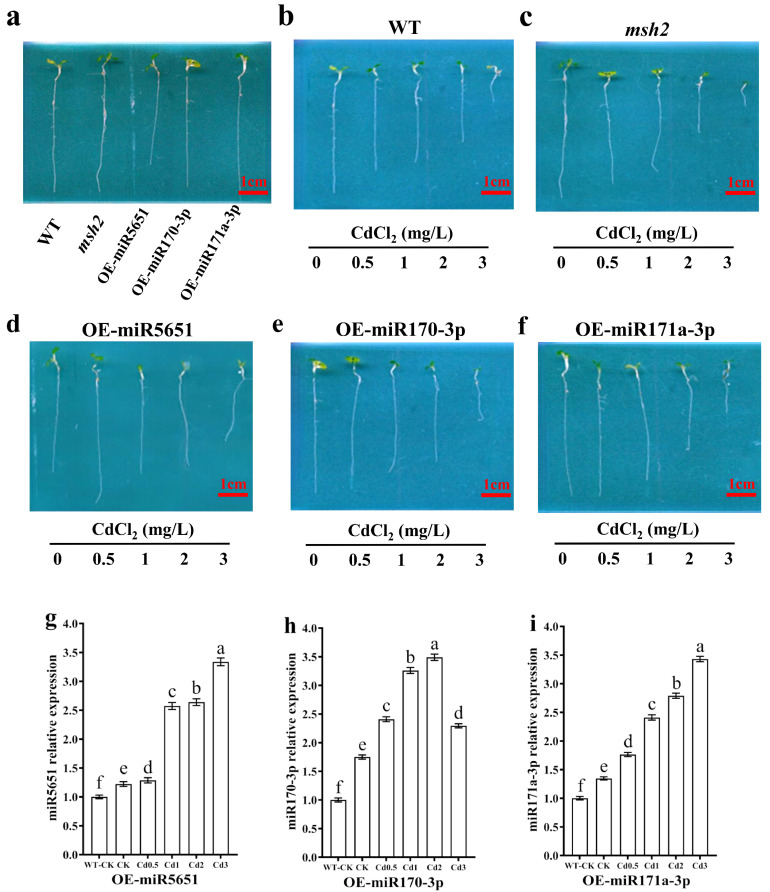
The effect of miRNAs targeting and regulating *AtMSH2* on plant growth under Cd stress. (**a**) The phenotype of WT, *Atmsh2*, OE-miR5651, OE-miR170-3p, and OE-miR171a-3p under the normal culture condition. The phenotype of WT (**b**), *Atmsh2* (**c**), OE-miR5651 (**d**), OE-miR170-3p (**e**), and OE-miR171a-3p (**f**) under the 0.5–3 mg/L Cd treatments. The relative expression levels of miR5651 (**g**), miR170-3p (**h**), and miR171a-3p (**i**) in their corresponding OE-miRNAs plants under the 0.5–3 mg/L Cd treatments. The genes expression levels in the control (WT-CK) were set to 1 as the normalization for qRT-PCR analysis using the operational formula 2^−ΔΔCt^. Data are shown as mean ± SD of three independent experiments, each biological replicate with three technical assays. Different letters indicate statistically significant differences between treatments at *p* < 0.05 by one-way ANOVA with Tukey’s test.

To explore the effect of miRNAs targeting and regulating *AtMSH2* on the *Arabidopsis* plants growth under Cd stress, the WT, *Atmsh2*, OE-miR5651, OE-miR170-3p and OE-miR171a-3p plants were subjected to different Cd concentration stress for 5 days. As showed in Figure 2a, there was no significant difference on the plant growth between the WT, *Atmsh2*, OE-miR170-3p, and OE-miR171a-3p under the normal condition, with an exception that OE-miR5651 plant roots are shorter than those lines. Compared with their respective control (0 Cd treatment), 0.5 mg/L Cd treatment could significantly inhibit WT and *Atmsh2* plant roots growth, and the inhibition gradually increased with the increase in Cd concentration (Figure 2b–f). However, 0.5 mg/L Cd treatment had no significant effect on the root growth of OE-miR170-3p and OE-miR171a-3p plants when compared with their respective control. In contrast, 0.5 mg/L Cd treatment could promote root growth in OE-miR5651 plants compared to the control. In addition, when the Cd concentration reached 1 mg/L, the degree of root growth inhibition increased with the increase in Cd concentration.

qRT-PCR results showed that the expression levels of miR5651, miR170-3p, and miR171a-3p in the OE-miR5651, OE-miR170-3p, and OE-miR171a-3p plants were upregulated by the Cd stresses with a dose-dependent relationship to the Cd concentration with an exception that the expression level of miR170-3p in the OE-miR170-3p plant roots under the Cd3 treatment was lower than that in the Cd1 and Cd2 treatment (Figure 2g–i).

**Figure 3:**
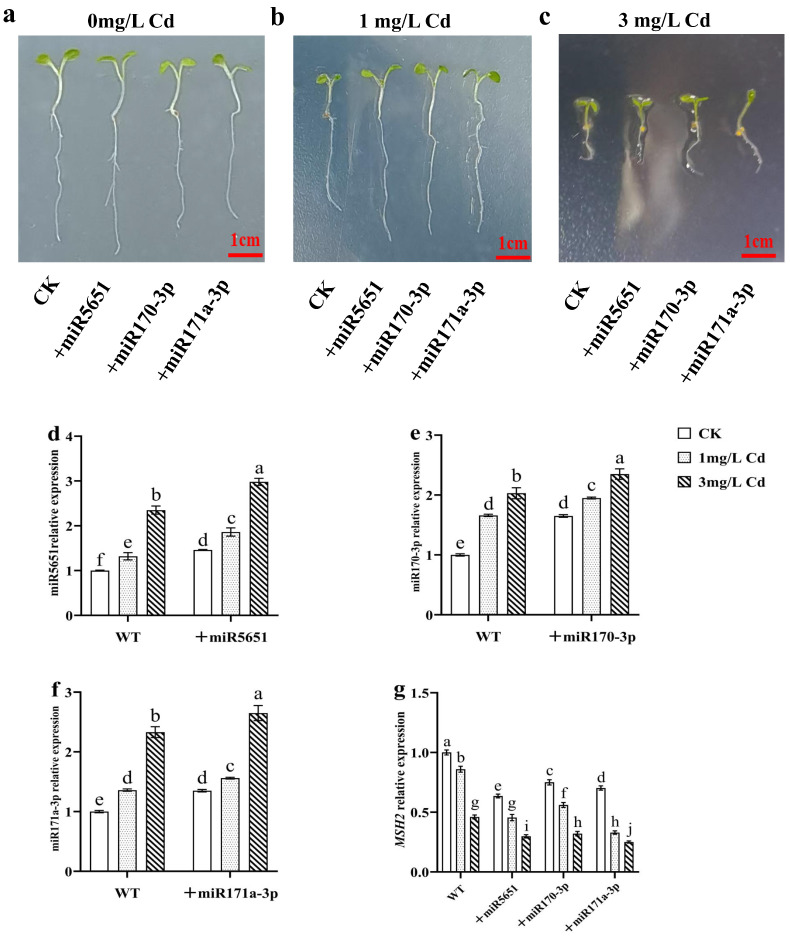
Exogenous miRNAs treatments on the plant growth of WT under Cd stress. (**a**–**c**) The effect of exogenous miRNAs treatments on the phenotype of WT under the Cd stress. The effect of exogenous miRNAs treatments on the relative expression levels of miR5651 (**d**), miR170-3p (**e**), miR171a-3p (**f**), and *AtMSH2* (**g**) in WT under the 0, 1, and 3 mg/L Cd treatments. The gene expression levels in the control (WT) were set to 1 as the normalization for qRT-PCR analysis using the operational formula 2^−ΔΔCt^. Data are shown as mean ± SD of three independent experiments, and each biological replicate with three technical assays. Different letters indicate statistically significant differences between treatments at *p* < 0.05 by one-way ANOVA with Tukey’s test.

As shown in Figure 3, exogenous miRNAs treatments had no significant effect on the plant growth of WT under normal growth conditions. As expected, exogenous miR5651, miR170-3p, and miR171a-3p treatments improved the Cd tolerance of WT plants. In addition, exogenous miRNAs treatments upregulated the expression levels of miR5651, miR170-3p, and miR171a-3p, and downregulated the expression levels of *AtMSH2* genes in the WT plant roots under the Cd stress, respectively.

### 3.4. The Effect of miRNAs Targeting and Regulating AtMSH2 on DNA Damage Response Signal Transduction Under Cd Stress

**Figure 4:**
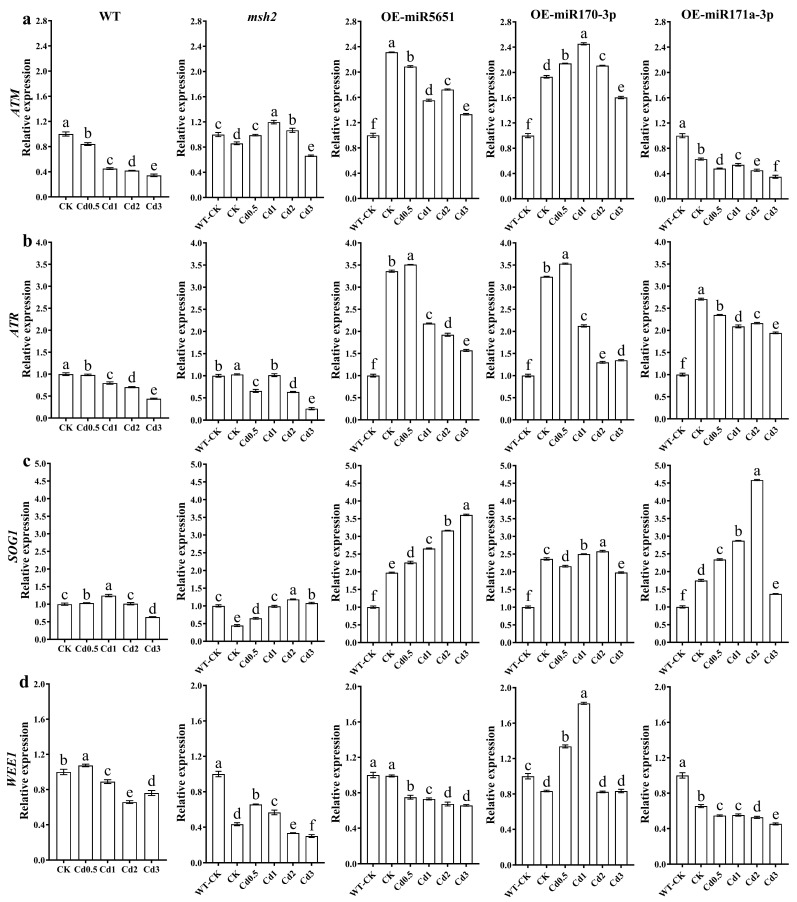
The effect of miRNAs targeting and regulating *AtMSH2* on DNA damage response signal transduction under Cd stress. The relative expression levels of *AtATM* (**a**), *AtATR* (**b**), *AtSOG1* (**c**), and *AtWEE1* (**d**) in the WT, *Atmsh2*, OE-miR5651, OE-miR170-3p, and OE-miR171a-3p plant roots under Cd stress. The gene expression levels in the control (WT-CK) were set to 1 as the normalization for qRT-PCR analysis using the operational formula 2^−ΔΔCt^. Data are shown as mean ± SD of three independent experiments, and each biological replicate with three technical assays. Different letters indicate statistically significant differences between treatments at *p* < 0.05 by one-way ANOVA with Tukey’s test.

To explore the effect of miR5651, miR170-3p, and miR171a-3p targeting and regulating *AtMSH2* on the DNA damage signal transduction in *Arabidopsis* plants under Cd stress, the relative expression levels of *AtATM*, *AtATR*, *AtSOG1*, and *AtWEE1* genes in WT, *Atmsh2*, OE-miR5651, OE-miR170-3p, and OE-miR171a-3p plant roots under Cd stress were determined by qRT-PCR. As shown in Figure 4, compared with the control (WT-CK), Cd stress dose-dependently downregulated the expression levels of *AtATM* and *AtATR* in the WT, *Atmsh2*, OE-miR5651, OE-miR170-3p, and OE-miR171a-3p plant roots, with the exception that 0.5–2 mg/L Cd treatment upregulated the expression levels of *AtATM* in the OE-miR170-3p. It is worth noting that the expression levels of *AtATM* and *AtATR* in OE-miR5651, OE-miR170-3p, and OE-miR171a-3p plant roots were significantly higher than those of WT and *Atmsh2* plant roots under the same Cd treatments. Furthermore, Cd treatments upregulated the expression levels of *AtSOG1* in these miRNA overexpression plant roots when comparted with their corresponding control, and had higher expression levels than those of WT and *Atmsh2*. In contrast, the expression levels of *AtWEE1* in OE-miR5651 and OE-miR171a-3p plant roots under Cd treatments were lower than those of WT, but higher than those of *Atmsh2*.

### 3.5. The Effect of miRNAs Targeting and Regulating AtMSH2 on DNA Mismatch Damage Repair Under Cd Stress

**Figure 5:**
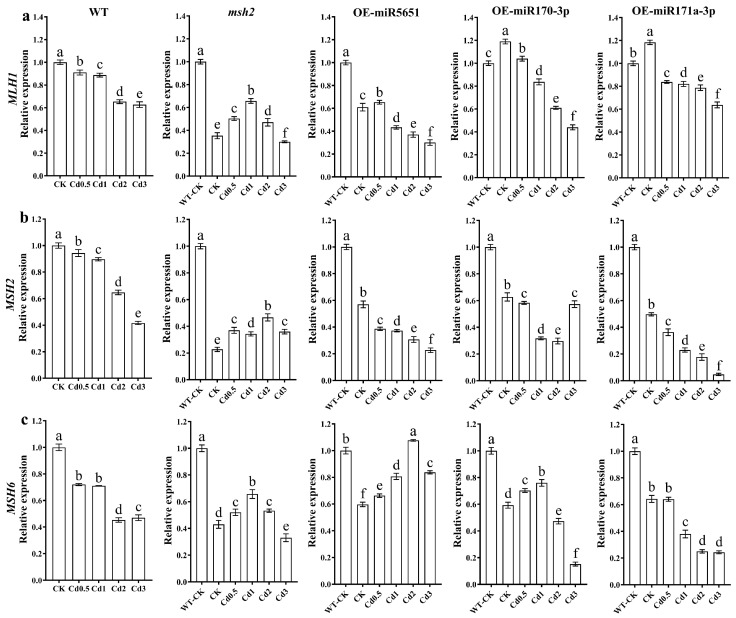
The effect of miRNAs targeting and regulating *AtMSH2* on DNA mismatch damage repair under Cd stress. The relative expression levels of *AtMLH1* (**a**), *AtMSH2* (**b**), and *AtMSH6* (**c**) in the WT, *Atmsh2*, OE-miR5651, OE-miR170-3p, and OE-miR171a-3p plant roots under Cd stress. The gene expression levels in the control (WT-CK) were set to 1 as the normalization for qRT-PCR analysis using the operational formula 2^−ΔΔCt^. Data are shown as mean ± SD of three independent experiments, and each biological replicate with three technical assays. Different letters indicate statistically significant differences between treatments at *p* < 0.05 by one-way ANOVA with Tukey’s test.

To explore the effect of miR5651, miR170-3p, and miR171a-3p targeting and regulating *AtMSH2* on the DNA damage mismatch damage repair in *Arabidopsis* plants under Cd stress, the relative expression levels of *AtMLH1*, *AtMSH2*, and *AtMSH6* genes in WT, *Atmsh2*, OE-miR5651, OE-miR170-3p, and OE-miR171a-3p plant roots under Cd stress were determined by qRT-PCR. As shown in Figure 5, compared with the control group (WT-CK), Cd stress dose-dependently downregulated the relative expression levels of *AtMLH1*, *AtMSH2*, and *AtMSH6* in WT plant roots. Compared with CK, 0.5–2 mg/L Cd treatment significantly upregulated the relative expression levels of *AtMLH1*, *AtMSH2*, and *AtMSH6* in *Atmsh2* plant roots, but 3 mg/L Cd treatment significantly downregulated the expression levels of these genes. Compared with CK, 0.5–3 mg/L Cd treatment significantly downregulated the relative expression levels of *AtMLH1* and *AtMSH2* in the root systems of OE-miR5651, OE-miR170-3p, and OE-miR171a-3p plants, with an exception that 0.5 mg/L Cd treatment significantly upregulated the expression level of *AtMLH1* in OE-miR5651. Compared with CK, Cd stress upregulated the expression level of the *AtMSH6* gene in OE-miR5651. In OE-miR170-3p, the expression level of the *AtMSH6* gene was upregulated by 0.5–1 mg/L Cd treatment and downregulated by 1–2 mg/L Cd treatment. In OE-miR171a-3p, the expression level of the *AtMSH6* gene was significantly downregulated by 0.5–3 mg/L Cd treatment.

### 3.6. The Effect of miRNAs Targeting and Regulating AtMSH2 on DNA HR and NHEJ Under Cd Stress

**Figure 6:**
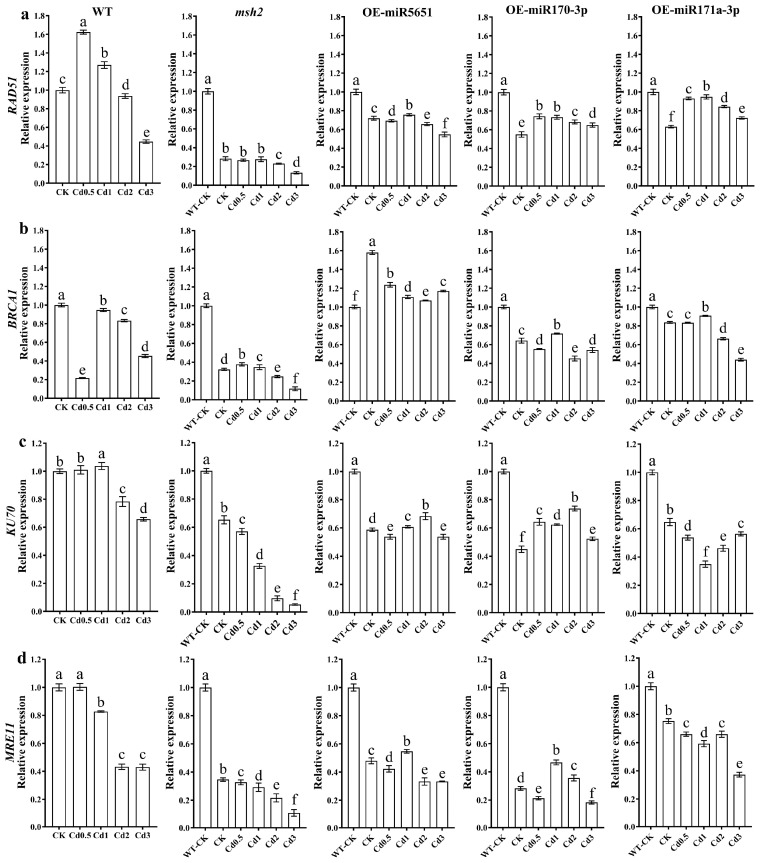
The effect of miRNAs targeting and regulating *AtMSH2* on DNA HR and NHEJ under Cd stress. The relative expression levels of *AtRAD51* (**a**), *AtBRCA1* (**b**), *AtKU70* (**c**), and *AtMRE11* (**d**) in the WT, *Atmsh2*, OE-miR5651, OE-miR170-3p, and OE-miR171a-3p plant roots under Cd stress. The gene expression levels in the control (WT-CK) were set to 1 as the normalization for qRT-PCR analysis using the operational formula 2^−ΔΔCt^. Data are shown as mean ± SD of three independent experiments, and each biological replicate with three technical assays. Different letters indicate statistically significant differences between treatments at *p* < 0.05 by one-way ANOVA with Tukey’s test.

To explore the effect of miR5651, miR170-3p, and miR171a-3p targeting and regulating *AtMSH2* on the homologous recombination (HR) and non-homologous end joining (NHEJ) of DNA damage repair in *Arabidopsis* plants under Cd stress, the relative expression levels of *AtRAD51*, *AtBRCA1*, *AtKU70*, and *AtMRE11* genes in WT, *Atmsh2*, OE-miR5651, OE-miR170-3p, and OE-miR171a-3p plant roots under Cd stress were determined by qRT-PCR. As shown in Figure 6, compared with the WT-CK, 2–3 mg/L Cd treatment significantly downregulated the expression levels of *AtRAD51*, *AtBRCA1*, *AtKU70*, and *AtMRE11* genes in WT plant roots. However, 0.5–1 mg/L Cd treatment upregulated the expression level of *AtRAD51* gene in WT, but downregulated the expression level of *AtBRCA1* gene, and showed no significant effect on the expression level of *AtKU70*. The expression levels of *AtRAD51*, *AtKU70*, and *AtMRE11* in OE-miR5651, OE-miR170-3p, and OE-miRNA171a-3p plant roots under 0.5–2 mg/L Cd treatments were significantly lower than those of WT, but higher than those of *Atmsh2*. Under Cd treatments, the expression level of *AtBRCA1* in the roots of OE-miR5651 plants was the highest among all genotypes, while that in *Atmsh2* roots was the lowest.

### 3.7. The Effect of miRNAs Targeting and Regulating AtMSH2 on Cell Cycle Regulation Under Cd Stress

**Figure 7:**
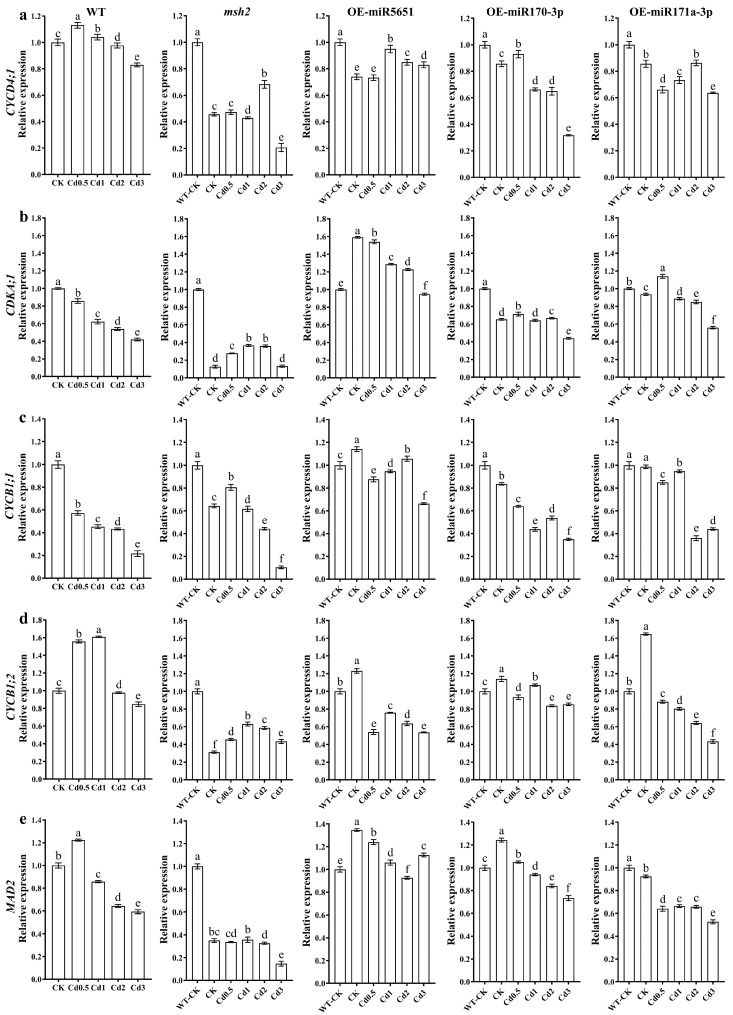
The effect of miRNAs targeting and regulating *AtMSH2* on cell cycle regulation under Cd stress. The relative expression levels of *AtCYCD4;1* (**a**), *AtCDKA;1* (**b**), *AtCYCB1;1* (**c**), *AtCYCB1;2* (**d**), and *AtMAD2* (**e**) in the WT, *Atmsh2*, OE-miR5651, OE-miR170-3p, and OE-miR171a-3p plant roots under Cd stress. The gene expression levels in the control (WT-CK) were set to 1 as the normalization for qRT-PCR analysis using the operational formula 2^−ΔΔCt^. Data are shown as mean ± SD of three independent experiments, and each biological replicate with three technical assays. Different letters indicate statistically significant differences between treatments at *p* < 0.05 by one-way ANOVA with Tukey’s test.

To explore the effect of miR5651, miR170-3p, and miR171a-3p targeting and regulating *AtMSH2* on the cell cycle regulation of *Arabidopsis* plants under Cd stress, the relative expression levels of *AtCYCD4;1*, *AtCDKA;1*, *AtCYCB1;1*, *AtCYCB1;2*, and *AtMAD2* genes in WT, *Atmsh2*, OE-miR5651, OE-miR170-3p, and OE-miR171a-3p plant roots under Cd stress were determined by qRT-PCR. As shown in Figure 7, 1–3 mg/L Cd treatments downregulated the expression levels of *AtCYCD4;1*, *AtCDKA;1*, *AtCYCB1;1*, *AtCYCB1;2*, and *AtMAD2* genes in the WT plant roots, with an exception that 1 mg/L Cd treatments upregulated the expression of *AtCYCB1;2*. Under Cd treatments, the expression level of *AtCYCD4;1*, *AtCDKA;1*, *AtCYCB1;1*, *AtCYCB1;2*, and *AtMAD2* in the roots of OE-miR5651, OE-miR170-3p, and OE-miR171a-3p plants was higher than that in *Atmsh2* roots. In addition, the expression level of *AtCDKA;1*, *AtCYCB1;1*, and *AtMAD2* in the miRNA overexpression plant roots under Cd treatments was higher than that in the WT.

## 4. Discussion

MiR5651, miR170-3p, and miR171a-3p were found as underlying regulators to regulate of *AtMSH2* based on bioinformatics prediction and our preliminary studies using miRNA sequencing. Building on our previous finding that *MSH2* responds to Cd stress, we further observed Cd-responsive expression of these miRNAs. Moreover, overexpression of the above miRNAs promoted Cd tolerance of *Arabidopsis*. Both elucidating how these three miRNAs target the *AtMSH2* gene to modulate DDR pathways and clarifying the mechanisms underlying the Cd resistance phenotype, constitute the primary objectives of this study.

### 4.1. miR5651, miR170-3p, and miR171a-3p Downregulate AtMSH2 Expression but Do Not Impair MMR-Mediated DDR

In this study, miR5651, miR170-3p, and miR171a-3p were first validated to target *AtMSH2* by tobacco dual-luciferase reporter systems in *vitro* and *Arabidopsis* transgenic lines in vivo. In miRNA-overexpression transgenic lines, *AtMSH2* expression was significantly suppressed by the above three miRNAs. However, the suppression level is lower than that observed in *Atmsh2*, with comparatively minor effects on *MLH1* and *MSH6* expression. Although the knockdown effect of miRNAs at the post-transcriptional level is inferior to T-DNA insertion mutants that knock out target genes at the DNA level, it is an effective approach to regulate the expression of target genes [50]. Nonetheless, multiple regulatory pathways, including DNA level, transcriptional level, post-transcriptional level, translational level, and post-translational level, exist within cells to control gene expression. As an epigenetic regulatory mechanism, miRNAs can only partially regulate gene expression and cannot fully govern its downstream functions and signaling pathways [51,52]. Therefore, overexpression of these miRNAs cannot substantially suppress *AtMSH2* expression or significantly impair the associated *MLH1* and *MSH6* genes, thereby weakening the MMR-mediated DDR.

MMR-mediated DDR is crucial for Cd-induced DNA damage, which involves MSH2, MSH6, MSH3, and MSH7 that form MutS homolog complexes that recognize base–base mismatches, insertion/deletion loops and interstrand cross-links. When MMR-mediated DDR is activated by Cd-induced DNA damage, HR repair is recruited, and cell cycle arrest occurs until lesions are repaired [16]. *ATM* and *ATR* are the key protein kinases, activate thousands of transcriptional factors that respond to mismatches, SSBs, and DSBs induced by DDR. DNA lesions like mismatches and SSBs predominantly trigger ATR-dependent DDR, and activation of *ATM* is usually responsible for DSBs. In this study, compared with WT and *Atmsh2*, *AtATR* and *AtATM* expression was significantly promoted, and HR and NHEJ repair were not significantly suppressed. Furthermore, cell cycle arrest primarily occurred at the G_2_ phase, driven by upregulated mitotic checkpoint *AtMAD2* and both stably expressed *AtCYCD4;1* and *AtCDKA;1* responsible for G_1_-S transition, which suggested that MMR-mediated DDR remained functional despite miRNA-mediated *AtMSH2* downregulation. Phenotypic and gene expression analyses revealed that transgenic plants overexpressing candidate miRNAs (miR5651, miR170-3p, and miR171a-3p) exhibited sustained robust expression of *AtRAD51* and *AtBRCA1*, whereas these DNA repair genes were significantly downregulated in *Atmsh2* mutants. Collectively, our findings demonstrate that miR5651, miR170-3p, and miR171a-3p enhance *Arabidopsis* tolerance to Cd stress through fine-tuned modulation of stress-responsive pathways, rather than complete suppression of *AtMSH2* expression.

### 4.2. miR5651, miR170-3p, and miR171a-3p Promote Cd Tolerance Due to Multiple DDR Engagement

According to the phenotype of overexpressed miRNA transgenic lines exposed to Cd and Cu stress, the above three miRNAs could promote *Arabidopsis* Cd tolerance compared with WT and *Atmsh2*. With the increasing gradient of Cd concentration, the root growth reduction in overexpressed miRNA seedlings was mitigated compared with WT seedlings. Heavy metal stress inevitably leads to reactive oxygen species (ROS) and damage to nucleic acids, proteins, and lipids. Cd stress primarily induces DNA damage, a critical cellular injury, which is one of the primary culprits responsible for growth inhibition. When DNA damage happens, cell cycle arrest will be triggered to maintaining genome stability and replication accuracy. G_1_/S and G_2_/M arrest are common responses to DNA lesions induced by heavy metals, whereas G_2_/M arrest supports plant growth potential. There are two convincing reasons to explain why G_2_/M arrest is better than G_1_/S for plants exposed to heavy metals stress. On the one hand, G_1_/S arrest will cause the comprehensively stationary state of cell reproduction. Based on this state, cell morphology remains unchanged, which suggests that plant cells will not enlarge. Furthermore, plant growth retardation is observed at the whole plant level, which is usually assumed to be stress sensitive [21]. On the other hand, the mitosis will activate until DNA damage is repaired. Although multiple DNA repair pathways exist from G_1_ to G_2_ phase, G_2_ phase is preferred by more error-free repair including nucleotide excision repair (NER), base excision repair (BER), MMR, and HR repair. Therefore, when cell cycle is arrest at G_2_/M phase due to DNA damage, DNA repair is efficiently driven through DDR, leading to the following mitosis after lesions repaired. Also, since the finished DNA replication and promoted synthesis of mRNAs and proteins, cell volume will increase for preparations of M phase. Thus, compared with at G_1_/S arrest, plants at G_2_/M phase are assumed to be more tolerant for stress.

In *Atmsh2*, *AtMSH2* expression was significantly downregulated due to T-DNA insertion in the promoter region of the *AtMSH2* gene, leading to the severe impairment of MMR function. MMR disorder further resulted in DDR switching, resulting in a transition from G_2_/M to G_1_/S arrest [18]. Therefore, stress intolerance was observed in *Atmsh2* when exposed to Cd. In miRNA-overexpressed transgenic lines, the miRNAs partially suppressed *AtMSH2* expression while retaining mismatch recognition, whereas MMR-mediated DDR maintained functional due to elevated *AtATR* expression and stably expressed *AtRAD51* and *AtBRCA1*, which suggests functional MMR-mediated DDR [17]. However, both significantly expressed *AtATR* and *AtATM* indicated the activation of other multiple DDR, recruiting multiple DNA repair pathways. The underlying process can accelerate DNA damage repairing and finish DDR, leading to the mitosis. Thus, the engagement of multiple DDR promotes plant Cd tolerance in miRNAs-overexpressed transgenic lines because of limited knockdown effect on *AtMSH2*.

### 4.3. miR5651, miR170-3p, and miR171a-3p Are Capable to Induce Plant-to-Plant Cd Tolerance

Wild-type *Arabidopsis* seedlings acquired Cd tolerance after exogenous application of miR5651, miR170-3p, and miR171a-3p. This observation supports the role of these miRNAs in enhancing Cd stress tolerance and suggests that plants tolerant to Cd overexpressing these miRNAs may improve the resistance to Cd in neighboring plants via miRNA transfer between plants. In oncology, miRNAs serve as metastasis biomarkers and mediate distant cellular communication [52], whereas in plants, they act as signaling molecules that enable gene silencing across species and stress adaptation [24,53]. Although detailed mechanisms of miRNA entry into plant cells remain incompletely resolved, endocytosis and pinocytosis facilitate transmembrane transport, a process documented in animal studies where miRNAs originating from plants, including representative examples such as miR168a, traverse mammalian intestinal barriers via sequential transepithelial transport, ultimately regulating liver gene expression [54,55]. Similarly, miRNAs from plants, specifically rice (*Oryza sativa* L.) miR159a.1-1 and miR167a, enter insect epithelia, modulating *PLCβ* and *RdRp* expression [56]. The exogenous miRNA application experiment was conducted as a supplementary investigation to our prior mechanistic research, assessing the phytoremediation potential of this approach. Given inherent efficiency limitations in exogenous miRNA transmission and greater complexity of regulatory processes in natural systems, we selected 0, 1, and 3 mg/L Cd^2+^ concentrations rather than a full gradient for validation. Intercellular transport is further evidenced by miRNA trafficking mediated by phloem or xylem through plasmodesmata [57,58,59,60,61], with grafting experiments confirming transfer of miR166a and miR395b from rootstock to scion to regulate sulfur metabolism in tomato (*Solanum lycopersicum* L.) [41,62,63,64].

Plant miRNAs exhibit remarkable stability extracellularly. For instance, miR2911 from honeysuckle (*Lonicera japonica* Thunb.) maintains antiviral activity even after boiling [65]. In this study, chemically synthesized exogenous miRNAs delivered without RNA-binding proteins effectively enhanced Cd tolerance in *Arabidopsis*. This demonstrates that plant miRNAs possess intrinsic signaling capacity independent of protective complexes. While this study used exogenous application, endogenous miRNA secretion via extracellular vesicles provides a natural pathway for communication between plants [66]. These insights suggest viable strategies for phytoremediation and crop breeding: engineering plants that secrete miRNAs could confer tolerance across entire fields to Cd, enhancing decontamination efficiency while reducing risks of transgene dispersal.

## 5. Conclusions

In this study, miR5651, miR170-3p, and miR171a-3p targeting *AtMSH2* were validated using the dual-luciferase reporter systems in vitro, followed by transformation with miRNAs in vivo. The qRT-PCR revealed that these miRNAs exhibited dose-dependent upregulation under Cd stress. However, based on the plant growth under Cd stress, miRNA-overexpressed mutants displayed enhanced Cd tolerance. Furthermore, this observation was further supported by exogenous application of these miRNAs to wild-type *Arabidopsis*, suggesting the miRNAs transferring and mediating the nearby plants in a plant-to-plant manner. Mechanistically, overexpression of these miRNAs activated ATR- and ATM-dependent DDR, inducing G_2_/M arrest to allow error-free repair. Notably, *AtRAD51* and *AtBRCA1* expression remained stable, ensuring HR efficiency despite partial suppression of *AtMSH2* function. The partial suppression of *AtMSH2* preserved MMR function, while co-activation of multi-pathway DDR engagement, enhancing Cd tolerance. This study provides a novel principle for elucidating Cd tolerance and offers insights into Cd tolerance breeding. However, the current research remains at the laboratory-based theoretical exploration stage, and its practical efficacy in authentic Cd-contaminated soil and aquatic environments requires validation. Further development of this study will accelerate the practical application of Cd phytoremediation technologies, while enhancing the efficiency of crop breeding under Cd stress and providing novel genetic resources and regulatory targets for Cd-tolerant germplasm innovation.

## Figures and Tables

**Table 1:** Prediction candidate miRNAs targeting the *AtMSH2*.

miRNA ID	miRNA Sequence	Target Sequence	Target Gene
ath-miR5651	TTGTGCGGTTCAAATAGTAAC	ATAACTATGGGAACTTCACAA	*AtMSH2*
ath-miR170-3p	TGATTGAGCCGTGTCAATATC	CTTACTGCCTTGGCTCAAGCA	*AtMSH2*
ath-miR171a-3p	TGATTGAGCCGCGCCAATATC	CTTACTGCCTTGGCTCAAGCA	*AtMSH2*

## Data Availability

The original contributions presented in the study are included in the article/Supplementary Material, further inquiries can be directed to the corresponding author.

## Supplementary Materials

The following supporting information can be downloaded at https://www.mdpi.com/article/10.3390/plants14132028/s1. Table S1: Primers used in genetic transformation, dual-luciferase assay, and qRT-PCR analysis.
